# Artificial Intelligence as a Catalyst for Antimicrobial Discovery: From Predictive Models to De Novo Design

**DOI:** 10.3390/microorganisms14020394

**Published:** 2026-02-06

**Authors:** Romaisaa Boudza, Salim Bounou, Jaume Segura-Garcia, Ismail Moukadiri, Sergi Maicas

**Affiliations:** 1 Engineering School in Biomedical and Biotechnology, Euromed University of Fes, Eco-Campus UEMF, Route de Meknes (RN6, Rond-Point Bensouda), Fez 30070, Morocco; r.boudza@ueuromed.org (R.B.); s.bounou@ueuromed.org (S.B.); 2 Department of Microbiology and Ecology, Faculty of Biology, Universitat de València, 46100 Burjassot, Spain; 3 Faculty of Pharmacy, Euromed University of Fes, Eco-Campus UEMF, Route de Meknes (RN6, Rond-Point Bensouda), Fez 30070, Morocco; 4 Department of Computer Science, School of Engineering, Universitat de València, 46100 Burjassot, Spain; jaume.segura@uv.es

**Keywords:** antimicrobial resistance, artificial intelligence, antibiotic discovery, antimicrobial peptides, machine learning, deep learning, generative models, drug design, computational biology

## Abstract

Antimicrobial resistance represents one of the most critical global health challenges of the 21st century, urgently demanding innovative strategies for antimicrobial discovery. Traditional antibiotic development pipelines are slow, costly, and increasingly ineffective against multidrug-resistant pathogens. In this context, recent advances in artificial intelligence have emerged as transformative tools capable of accelerating antimicrobial discovery and expanding accessible chemical and biological space. This comprehensive review critically synthesizes recent progress in AI-driven approaches applied to the discovery and design of both small-molecule antibiotics and antimicrobial peptides. We examine how machine learning, deep learning, and generative models are being leveraged for virtual screening, activity prediction, mechanism-informed prioritization, and de novo antimicrobial design. Particular emphasis is placed on graph-based neural networks, attention-based and transformer architectures, and generative frameworks such as variational autoencoders and large language model-based generators. Across these approaches, AI has enabled the identification of structurally novel compounds, facilitated narrow-spectrum antimicrobial strategies, and improved interpretability in peptide prediction. However, significant challenges remain, including data scarcity and imbalance, limited experimental validation, and barriers to clinical translation. By integrating methodological advances with a critical analysis of the current limitations, this review highlights emerging trends and outlines future directions aimed at bridging the gap between in silico discovery and real-world therapeutic development.

## 1. Introduction

Throughout history, humanity has struggled with the devastating impact of bacterial infections. In 1900, pneumonia, tuberculosis, diarrhea, enteritis, and diphtheria accounted for around one-third of all deaths, with children under five years old representing 30.4% of these deaths [1]. Treatments for these infections were often unsafe and largely ineffective. Paul Ehrlich’s pioneering research in the early 20th century marked a turning point, as he aimed to develop a “magic bullet” capable of selectively targeting pathogens without harming the host [2]. This work led to the development of Salvarsan in 1910, the first effective chemotherapeutic agent for syphilis [3].

**Figure 1:**
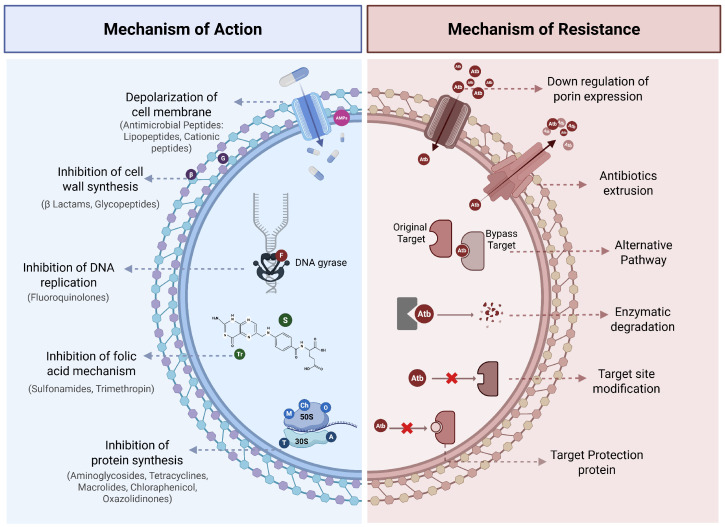
Mechanisms of action and mechanisms of resistance to antibiotics in bacteria. AMPs: Antimicrobial Peptides; Beta: Beta lactams; G: Glycopeptides; F: Fluoroquinolones; S: Sulfonamides; Tr: Trimethoprim; A: Aminoglycosides; T: Tetracyclines; M: Macrolides; Ch: Chloraphenicol; O: Oxazolidinones. Atb: Antibiotic. Created with: (https://BioRender.com/6qce679, accessed on 22 December 2025).

The accidental discovery of penicillin from the fungus *Penicillium notatum* by A. Fleming in 1928 marked the beginning of a new era in medicine by enabling targeted treatment for many infections [4]. Streptomycin, discovered by A. Schatz from the soil bacterium *Streptomyces griseus*, was later widely used against *Mycobacterium tuberculosis* [5]. These breakthroughs saved countless lives and significantly reduced mortality rates [1]. Subsequently, S.A. Waksman provided a comprehensive definition of antibiotics as substances produced by microorganisms that inhibit the growth of other microorganisms (bacteriostatic) or cause their death (bactericidal) [6,7]. Antibiotics act by interfering with essential bacterial processes, including the inhibition of cell wall synthesis [8], the disruption of cell membranes [9], the inhibition of DNA replication [10], protein synthesis [11], and folic acid metabolism [12] (Figure 1).

Since the widespread discovery and use of antibiotics, bacteria have developed smart strategies to evade their effects, driven by three powerful evolutionary measures: random mutations, selective pressure and horizontal gene transfer (HGT) [13,14]. Even without exposure to antibiotics, bacteria accumulate random mutations to improve their adaptability [15]. While some mutations are silent, some can alter the cellular targets of antibiotics, making them less effective [13]. The consumption of antibiotics eliminates susceptible bacteria while allowing the survival and multiplication of resistant ones, creating selective pressure [16]. However, bacteria do not only depend on random mutations; they actively exchange resistance genes through HGT [17]. These genetic adaptations help bacteria to evolve resistance mechanisms against antibiotics, including reduced permeability, active antibiotic efflux, target site modification or protection, enzymatic drug degradation and target bypass [18] (Figure 1).

*Enterococcus faecium*, *Staphylococcus aureus*, *Klebsiella pneumoniae*, *Acinetobacter baumannii*, *Pseudomonas aeruginosa*, and *Enterobacter* species are among the highly virulent bacteria known as ESKAPE pathogens. These bacteria were first recognized as critical multidrug-resistant pathogens for which the development of effective treatments was urgently required [19]. At present, antimicrobial resistance (AMR) represents one of the growing global health challenges of the 21st century. Globally, 4.95 million deaths were associated with bacterial resistance to antimicrobials in 2019. Of these, 1.27 million deaths were directly caused by AMR [20]. Current studies predict that AMR could lead in up to 10 million deaths each year by 2050, presenting a substantial threat to public healthcare systems and the global economy [20]. This underscores the urgent need for interventions; some of these interventions include the implementation of robust antibiotic stewardship programs to ensure proper clinical use, strengthening surveillance systems to monitor emerging resistance patterns, launching public health initiatives to raise awareness, and accelerating the development of novel antimicrobial therapies [21]. Particularly, the discovery of novel antimicrobial compounds is urgently needed, as many of the antibiotics currently in clinical use are no longer effective [22]. However, antibiotic discovery is a challenging, costly, and prone-to-failure process that can take ten years and costs hundreds of millions of dollars [23].

After the mid-20th century “Golden Age” of antibiotic discovery, innovation collapsed: since the 1980s, virtually no truly novel classes have reached the clinic, and only about five new classes have appeared since 2000. This historic success, driven by traditional paradigms, mining natural products and broad synthetic screens, is now exhausted. Most easily accessed microbial compounds have been repeatedly rediscovered, and high-throughput synthetic libraries have largely failed to yield new antibiotics. Even when promising hits emerge, development often fails due to pharmacological issues or poor drug properties, and economic/regulatory barriers [24]. These long-standing gaps, from technological limitations in discovery to translational obstacles in development, underscore the urgent unmet needs in antibiotic research and development. This highlights the necessity of innovative approaches in the search for antibiotics.

Artificial intelligence is emerging as a game-changing tool that offers new opportunities to deepen our understanding of resistance mechanisms, improve therapeutic approaches, and accelerate the development of novel antibiotics [25]. By integrating machine learning, deep learning, and experimental methodologies, AI enables researchers to analyze large datasets, predict resistance patterns, and design innovative treatment strategies with high accuracy and efficiency [26,27,28]. The application of AI in AMR research has the potential to transform how we combat resistant bacterial “superbugs” and to promote a more proactive and sustainable strategy for global health security. The purpose of this review is to address the following question: How can artificial intelligence approaches accelerate antimicrobial discovery, particularly regarding accuracy, efficiency, and novelty, and which AI models and computational tools are currently being applied? By analyzing original studies published between 2020 and 2025, this review also identifies knowledge gaps, discusses current challenges, and proposes future perspectives to optimize the integration of AI in antibiotic discovery. This review aims to serve as a reference for researchers working at the intersection of AI, microbiology, and drug discovery, fostering interdisciplinary collaboration to address the global AMR crisis.

## 2. Scope and Review Strategy

The information discussed in this review was selected through a structured and iterative search of major scientific databases, including PubMed, Web of Science, and Scopus. Selected pre-2020 references are included exclusively for historical and conceptual context and do not fall within the systematic review scope, which was restricted to AI-driven antimicrobial discovery studies published between 2020 and 2025. Rather than aiming for exhaustive coverage, we prioritized representative and high-impact experimental and computational works that illustrate key methodological advances, emerging trends, and translational challenges in AI-driven antimicrobial discovery.

### 2.1. Research Questions

- What artificial intelligence (AI) techniques are currently applied in antimicrobial discovery?
- Which AI algorithms demonstrate the highest effectiveness, and what factors contribute to their performance?
- What challenges limit the broader adoption of AI in antimicrobial discovery?
- How are generative AI frameworks used to design novel antibiotics and antimicrobial peptides (AMPs)?
- What AI-based tools exist for AMP prediction, and what are their accuracy, usability, and validation strategies?

### 2.2. Search Strategy

This section outlines the approach used to identify and collect information on the application of artificial intelligence in antibiotic discovery. An automated search was conducted to explore and retrieve the relevant scientific articles from three databases, using specific keywords developed to address the study’s research questions. The search terms used to construct the search strings were selected based on their common usage in previous manuscripts and their relevance to this research (artificial intelligence, machine learning, deep learning, generative AI, predictive AI, antimicrobials, antibiotics, drugs, antimicrobial resistance The following keyword strings were mapped out and applied for each database: (‘antimicrobial resistance’ OR ‘resistant bacteria’ OR ’Resistant microorganisms’) AND (‘artificial intelligence’ OR ‘deep learning’ OR ‘machine learning’ OR ‘generative AI’) AND (‘antibiotic discovery’ OR ‘drug discovery’ OR ’novel drug’). The 2020–2025 publication window was selected to capture the rapid evolution of deep learning, graph-based, and generative AI methodologies that currently define the state of the art in antimicrobial discovery, while earlier landmark studies were cited selectively for contextual background. The search strings employed are summarized in Table 1.

### 2.3. Selection of Records

During the initial screening stage, duplicate papers were eliminated. The remaining records underwent title and abstract screening to exclude reviews, reports, lecture notes, book chapters, and any other papers outside of journals. By limiting the focus to peer-reviewed journal articles, this step enhances the credibility of the selected contributions. Additionally, a secondary screening was conducted by applying predefined eligibility (inclusion and exclusion) criteria aligned with the review objectives. By using these criteria, it was ensured that only representative studies were included in this review. Both screenings were performed using the Rayyan software [29].

### 2.4. Inclusion Criteria

- Articles related to antimicrobial resistance.
- Relevant content on artificial intelligence algorithms for developing novel antibiotics.
- Publications in peer-reviewed scientific journals.
- Research articles with full-text accessibility.

### 2.5. Exclusion Criteria

- Research papers published before 2020.
- Research papers not related to the application of AI in antibiotic discovery.
- Non-English publications.
- Review papers, conference abstracts, or book chapters.

### 2.6. Quality Assessment (QA)

After the secondary screening, the selected papers underwent a quality assessment test to demonstrate the significance of the reviewed papers to the work’s contribution. The following four QA questions were used as a baseline screening framework to ensure the relevance, methodological transparency, and experimental support of the selected studies. Additional dimensions related to computational rigor and biological translatability were subsequently evaluated qualitatively during the thematic synthesis.

- Relevance of the study: Does the paper’s topic connect to AI-based antibiotic discovery?
- Clarity of the methodology: Is the research methodology properly described?
- Presentation of results: Does the publication include the results?
- Experimental validation and biological relevance: Does the paper include in vitro validation, and where applicable, evidence of in vivo evaluation, toxicity assessment, or pharmacokinetic considerations?

While the formal scoring of computational benchmarks and biological safety was not used as an exclusion criterion, aspects such as dataset curation, class imbalance handling, validation strategy (e.g., scaffold splitting or external testing), model interpretability, and downstream biological relevance (toxicity prediction, ADME profiling, and in vivo translation) were critically analyzed and discussed throughout the analysis and discussion sections. This qualitative approach was chosen to reflect the heterogeneity of AI methodologies and validation standards currently present in antimicrobial discovery research.

### 2.7. Data Extraction and Qualitative Synthesis

Data were extracted into a customized database, including information on study objectives and the methodology followed in each study. Given the heterogeneity in computational approaches, a narrative synthesis was considered the most appropriate approach for conducting the analysis. The findings are qualitatively summarized, with a focus on highlighting emerging trends in artificial intelligence-aided antimicrobial discovery.

To ensure methodological clarity, the included studies were analyzed and categorized according to three orthogonal dimensions: (i) AI model family (classical machine learning, deep learning, graph-based models, transformer architectures, and generative frameworks); (ii) input data modality (molecular graphs, physicochemical descriptors, protein or peptide sequences, structural binding-site representations, and multi-omic features); and (iii) antimicrobial target class (small-molecule antibiotics versus antimicrobial peptides, as well as broad-spectrum versus narrow-spectrum applications). These distinctions were used to contextualize downstream discovery performance, including predictive accuracy, chemical novelty, interpretability, and translational applicability, and are explicitly discussed throughout the Results sections. Across the reviewed literature, substantial heterogeneity was observed in dataset composition, annotation practices, and experimental protocols. Differences in antimicrobial activity thresholds, MIC reporting units, strain selection, and negative sample definition introduce variability that complicates direct comparison across studies. Moreover, many datasets remain limited in size and highly imbalanced, with active compounds or peptides representing a small fraction of the training data. These characteristics can inflate apparent model performance, particularly when random train–test splits are employed, and may limit generalization to novel chemical scaffolds, microbial species, or experimental conditions. While the present review does not impose formal dataset curation criteria as inclusion thresholds, recurring best practices emerge from high-performing and experimentally validated studies. These include careful dataset de-duplication, harmonization of activity annotations, explicit handling of class imbalance, the use of scaffold-based or external validation splits, and transparent reporting of negative examples. We highlight these practices throughout the synthesis and Discussion as informal benchmarks that can improve reproducibility and reduce failure modes in AI-driven antimicrobial discovery.

## 3. AI Strategies for Antimicrobial Discovery

Recent advances in artificial intelligence have reshaped multiple stages of the antimicrobial discovery pipeline, from early-stage virtual screening to de novo molecular and peptide design. Rather than constituting a single methodological framework, AI-driven antimicrobial discovery encompasses a diverse set of computational strategies tailored to different data modalities, antimicrobial classes, and translational objectives. In this section, we synthesize key AI approaches applied to the discovery of small-molecule antibiotics and antimicrobial peptides, emphasizing model architectures, data requirements, validation strategies, and emerging design paradigms. We highlight both convergent trends and unresolved challenges that define the current landscape of AI-enabled antimicrobial research.

### 3.1. Distribution of Selected Studies

**Figure 2:**
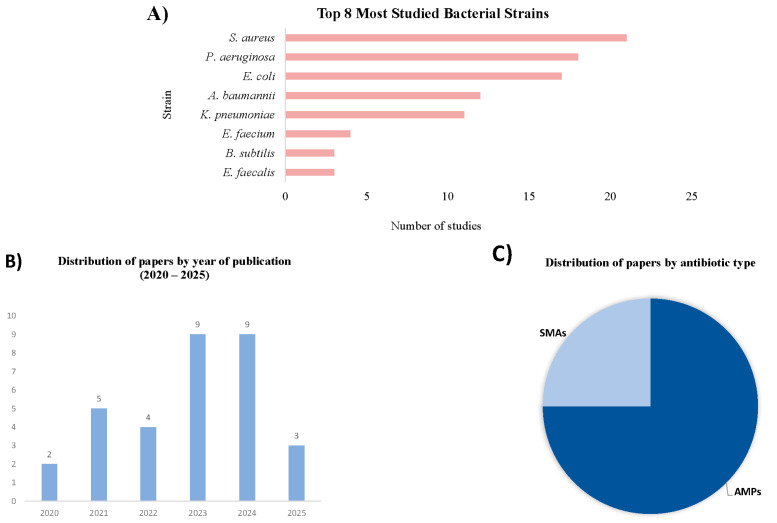
Characteristics of the included studies: (**A**) top 8 most-used bacterial strains; (**B**) distribution of papers by year of publication; (**C**) distribution of papers by antimicrobial type.

In this review, all the selected studies (*n* = 32) include in silico approaches. The analysis reveals a diverse set of antimicrobial types, with antimicrobial peptides representing the majority (75%) compared to small-molecule antibiotics (25%) (Figure 2).

**Figure 3:**
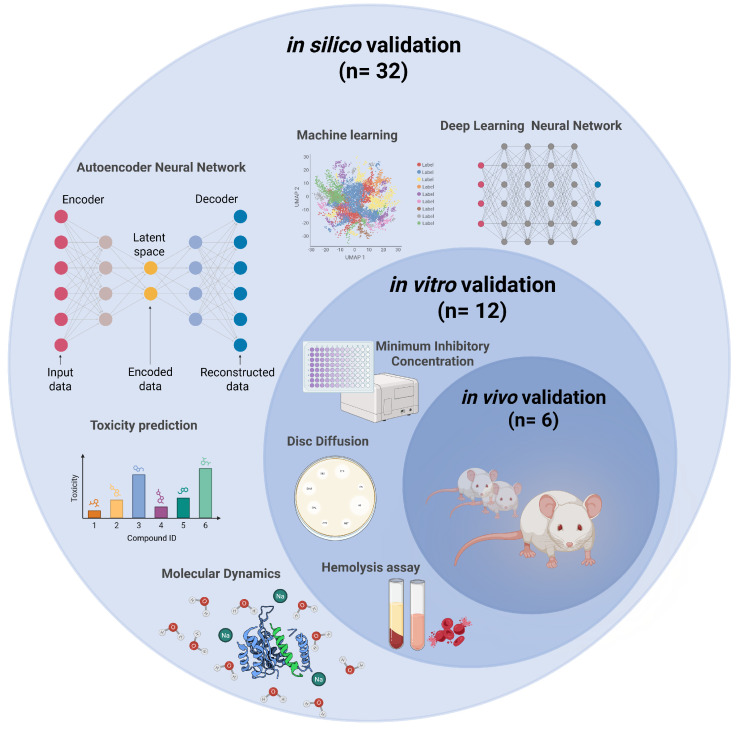
Overview of the validation level in AI-driven antimicrobial discovery: selected studies.

Among these 32 studies, 12 also included in vitro validation, and 6 of them extended to in vivo validation on murine models (Figure 3).

### 3.2. SMAs Discovery

There was heterogeneity regarding AI model architectures, optimization methods and their application. Predictive classifier models were by far the most common, appearing in 21 of the 32 studies (approximately 66%). These predictive approaches encompassed both traditional machine-learning classifiers and modern deep learning networks. Meanwhile, a smaller subset of studies explored generative AI models to generate novel antibiotic candidates.

#### 3.2.1. Predictive Models

A critical task in the early stages of antibiotic discovery is the identification of molecular compounds with potential antimicrobial activity. This task is conventionally framed as a classification problem, where the objective is to distinguish active molecules from inactive ones based on their structural and physicochemical characteristics. Recent advancements in artificial intelligence (AI), notably machine learning and deep learning, have markedly enhanced the accuracy and scalability of predictive models used in this domain. Among these, graph-based neural networks, particularly the Directed Message Passing Neural Network (D-MPNN), are gaining significant attention due to their ability to learn molecular features directly from graph-structural data like molecules, where atoms are represented as nodes and chemical bonds as edges [30]. However, training these models remains challenging due to the small and imbalanced available datasets. A limitation across benchmarking practices is the widespread reliance on random train–test splits, which can substantially overestimate model performance by allowing structurally similar compounds to appear in both training and test sets. More stringent strategies, such as scaffold-based splitting, temporal splits, or external validation on independent datasets, provide a more realistic assessment of generalization in antimicrobial discovery.

To overcome these limitations and improve predictive performance, these models are often combined with data augmentation techniques such as hybrid molecular representations that integrate learned graph embeddings with physicochemical descriptors extracted from tools like RDKit [30,31]. In addition, Bayesian hyperparameter optimization and ensembling-based augmentation techniques are frequently used to enhance model performance, mitigate overfitting and improve generalization across diverse chemical spaces. These strategies enabled large-scale virtual screening of millions of compounds and have successfully facilitated the discovery of both novel antibiotic candidates with a broad spectrum, such as halicin, and those with a narrow spectrum, such as abaucin [30,31].

Following this success, Rahman et al. (2022) extended the same model architecture to focus on *Burkholderia cenocepacia*, training it on a high-throughput dataset of 29,537 compounds [32]. By combining multitask learning with regression and binary classification, they boosted the predictive performance of the model. Scaffold-based splitting strategies were used to assess the model’s predictive performance on unseen chemotypes, providing a more realistic evaluation compared to random splits. Despite the slightly lower ROC-AUC of 0.823, the model effectively identified 22 novel compounds with activity against various ESKAPE pathogens [32]. In the same vein, recent strategies have extended beyond activity prediction to integrate a mechanism-prediction framework, combining machine learning with molecular docking, ADME profiling and pharmacokinetic filtering. These additional steps are important for enhancing the credibility and translational potential of AI-based predictions by supporting both mechanistic relevance and drug-likeness. Despite these advances, explicit explainability and interpretability frameworks are still inconsistently applied in AI-driven antimicrobial discovery. Techniques such as attention weight analysis, feature attribution methods (e.g., SHAP or Integrated Gradients), and emerging graph neural network interpretability tools have the potential to link model predictions to mechanistic hypotheses, improve biological inference, and enhance model trustworthiness. Their systematic adoption is likely to be essential for regulatory acceptance and for integrating AI predictions into mechanism-informed antimicrobial design pipelines.

Boulaamane et al. (2024) proposed a multi-model ensemble strategy that combines classical machine learning techniques that includes Random Forest, Support Vector Machine, K-Nearest Neighbors, Gaussian Naïve Bayes, with a Convolutional Neural Network (CNN) for quantitative structure–activity relationship (QSAR) modeling [33]. This study presented an effective framework for narrow-spectrum antibiotic discovery using a natural compound library targeting a specific mechanism of action (OmpW in *A. baumannii*). Although the dataset was curated for consistency in MIC units and duplicates removed, the study did not report applying preprocessing techniques such as noise filtering, class balancing, or redundancy correction, which constitute factors that are critical in enhancing model reliability [33]. Ultimately, the CNN model outperformed traditional ML methods and was used to identify desmethoxycurcumin as a promising OmpW inhibitor [33].

In a separate investigation, Wang et al. (2024) proposed a new workflow using a Latent Space Constraint Neural Network (LSCNN) coupled with dimensionality reduction techniques such as a Uniform Manifold Approximation and Projection (UMAP) algorithm to improve screening efficiency by targeting chemically diverse subsets from combinatorial libraries [34]. After being trained on this subset, LSCNN achieved a 60% hit rate, compared to only 5.3% when using random screening. Three purified hits showed significant activity (MIC = 12 µM), rapid bactericidal kinetics, and reduced resistance development, demonstrating strong consistency between in silico predictions and experimental outcomes [34].

More recently, Olayo-Alarcón et al. (2025) introduced a self-supervised graph neural network architecture called MoIE, which was pre-trained on 100,000 unlabeled compounds using the Barlow-Twins objective [35]. This pre-training step was intended to improve the generalizability of the model to low-resource tasks. The MoIE representations were then used alongside an XGBoost classifier to predict antimicrobial potential, showing improved accuracy compared to conventional ECFP4 fingerprints [35]. Detailed characteristics of the predictive models and key outcomes are compiled in Table 2.

While these models show strong performance in virtual screening and compound prioritization, several limitations persist. Many approaches depend on large, well-annotated datasets, which are often unavailable for rare pathogens. Some studies also neglect the key preprocessing steps needed to address chemical redundancy, class imbalance, and feature noise. Model generalizability to structurally diverse chemical spaces remains challenging, especially in real-world settings with novel compounds. Moreover, validation is frequently limited to in vitro or small-scale in vivo studies, requiring broader experimental confirmation before clinical translation.

#### 3.2.2. SMAs De Novo Design

According to recent artificial intelligence research, ML algorithms can auto-generate drug-like molecules. De novo drug discovery has been revolutionized by generative models, rendering the exploratory approach more efficient. Many models represent molecules using SMILES representations, framing molecule generation as a sequence-to-sequence learning task. While effective, this strategy necessitates large-scale pretraining and is sensitive to syntactic errors, which can generate invalid SMILES that cannot be converted to a chemical structure.

To solve this novel generative framework, Molecular Design via Attribute-Guided Search (MDAGS) was developed by combining a directed message-passing neural network (Chemprop) to encode molecular graphs with a GPT (Generative Pre-trained Transformer) model to generate the corresponding molecular SMILES sequence, thus generating novel potent antibiotic molecules with expected antibacterial activity [36]. The model was trained on a diverse set of antibiotic and natural products to learn a latent inhibition space optimized for antibacterial activity.

The GPT generator achieved high performance compared to conventional models in novelty and uniqueness measures by producing novel antibiotic candidates with significant structural diversity and improved predicted potency through the use of a guided search and swarm optimization within the latent inhibition space [36]. This method was based on graph-to-sequence generation to combine the strengths of both SMILES and graph representations, while avoiding their limitations.

Soon after this, Krishnan et al. (2023) developed a structure-based de novo drug design approach that combine a Graph Attention–Variational Autoencoder (GAT-VAE) model, trained on graph representations of protein binding sites, and a SMILES-VAE, trained on SMILES representations of millions of chemical structures [37]. This conditional molecule generator, optimized by reinforcement learning and a Drug–Target Affinity (DTA) model, was able to generate novel compounds with high similarity to known anti-tuberculosis agents, new scaffolds with preserved pharmacophoric features and strong predicted binding [37]. The use of only 37 molecules as a validation dataset and the lack of wet-lab validation limits the robustness of the evaluation, making it challenging to fully assess the generalizability and leaving the actual biological activity unconfirmed. Table 3 provides a structured synthesis of these generative frameworks, their datasets, performance metrics, and the results, emphasizing both their potential in de novo antibiotic design.

While both models generated novel molecules, the MDAGS approach offers greater flexibility and diversity in candidates, whereas the GAT-VAE with SMILES-VAE model is powerful for target-specific molecule design but requires further validation. In silico models based solely on antimicrobial activity or binding affinity may yield compounds with poor pharmacokinetic profiles and high toxicity, which are major causes of failure in clinical drug development. De novo molecules must undergo toxicity and ADME (Absorption, Distribution, Metabolism, and Excretion) evaluation to enhance their translational potential. This can be achieved through predictive models, multitask learning, or transfer learning to improve safety, efficacy, and pharmacokinetics.

Despite their ability to generate novel scaffolds, generative AI frameworks face practical constraints that limit their translational impact. A major challenge is retrosynthetic tractability, as many AI-designed molecules are difficult to synthesize. Although neural-network-based retrosynthesis tools and accessibility scores are being integrated, manufacturability and ADME/Tox liabilities remain insufficiently addressed. Most workflows rely on limited in silico filters, underscoring the need for multi-objective models that jointly optimize antimicrobial activity, synthetic feasibility, and pharmacokinetic properties to better support experimental development.

The experimental validation of AI-derived antimicrobial candidates remains largely confined to simplified in vitro assays, which often fail to capture host–pathogen interactions, immune modulation, and pharmacodynamic complexity. The absence of standardized experimental evaluation pipelines hampers the cross-study comparability and benchmarking of AI performance. Advanced validation platforms, including organoid-based systems and systematic in vivo models, are still rarely employed, representing a critical gap in the current translational ecosystem.

### 3.3. Analysis of Models for SMA Discovery

Reproducibility remains a major challenge in AI-driven antimicrobial discovery due to the frequent use of closed-source models and non-public datasets, and the incomplete reporting of hyperparameters, validation schemes, and computational workflows. Greater transparency in code availability, data curation, and benchmarking practices is essential to enable independent validation, fair model comparison, and translational reliability. Table 4 summarizes AI models used in SMAs discovery, highlighting their key advantages and limitations.

#### 3.3.1. Directed Message-Passing Neural Network (D-MPNN)

D-MPNN is an emerging type of Graph Neural Network (GNN) specifically developed to learn to predict molecular features from graph-structural data like molecules, where atoms are nodes and chemical bonds are edges [30]. Unlike standard GNNs, the distinct characteristic of this model is that an edge is the source and receiver of messages; instead of sending messages between atoms, messages are sent between bonds, and each bond has a direction [38]. This structure is special because it prevents the information of a message from going back and forth endlessly between two atoms, reducing noise and helping the network learn better representations. The model applies several message passing steps, during which it aggregates information from neighboring atoms and bonds to form a detailed representation of the local chemical environment [30]. In the readout phase, the hidden states of atoms in a molecule are aggregated to form a latent vector that encodes the whole molecule’s structure and characteristics [32].

In order to minimize prediction errors regarding molecular properties, hyperparameters like the hidden layer sizes, number of message-passing steps, and dropout are optimized using Bayesian optimization or grid search [30,32]. However, D-MPNNs are supervised learners; they require large, high-quality datasets with labeled outputs. For antimicrobial discovery, there is no sufficiently large, diverse public dataset to train D-MPNNs for general use. So, researchers have to build custom datasets through resource-intensive experimental screening [35]. Even with a custom-trained D-MPNN, the model may perform poorly on new microbial species or classes of molecules if they were not well represented in the training data. Training useful D-MPNNs often demands experimental screening campaigns of thousands of compounds, which is costly and time-consuming, limiting scalability.

#### 3.3.2. GPT Generator

GPT (Generative Pretrained Transformer), part of the family of large language models (LLM), is a model based on a transformer architecture adjusted from natural language processing for SMILES strings generation [39]. The model is composed of multiple stacked decoding blocks, each comprising a self-attention layer followed by a set of fully connected layers. It also employs a masked self-attention mechanism to learn the dependencies across the sequence, and ensures the model generates chemically valid SMILES step-by-step [36]. However, the model can generate duplicate or invalid structures due to the complexity of string-based generation.

#### 3.3.3. Graph Attention–Variational Autoencoders

GAT-VAE, a type of variational autoencoder developed for graphs, employs graph attention networks (GAT) in the encoder, and is suitable for capturing spatial relationships and high-dimensional omic features. The GAT-VAE encoder includes multi-head graph attention layers to process node features, embedding the input into a 256-dimensional latent vector, which is passed to the GAT-VAE decoder. The decoder reconstructs the graph structure from the latent vector using fully connected layers. The model is often trained with reconstruction loss and KL divergence loss, and uses the Adam optimizer with learning rate scheduling and batch normalization for stability [37]. However, training these models is challenging and complex and often leads to the reconstruction of molecules similar to those in the training set. These models struggle to directly enforce specific property targets or explore beyond the chemistry encoded in the training data. A concise overview of these models, including their strengths and limitations, is summarized in Table 3.

### 3.4. AMPs Discovery

#### 3.4.1. AI in Multi-Omic Data Mining

Antimicrobial peptide (AMP) mining involves analyzing biological sequences to identify potential AMPs. Artificial Intelligence-based techniques revolutionize in silico bioprospecting by allowing scientists to comprehensively and rapidly mine the proteome, genome or transcriptome of any organism for potent bioactive compounds. For example, in silico mining strategies have been applied to proteomes from multiple species, combining PepMultiFinder tool to find peptides matching strict physicochemical characteristics, using the CAMP3 machine learning platform that predicts AMP using SVM, RF, ANN, and DAC classifiers [40]. They successfully identified 11 novel antimicrobial peptides that were validated in vitro as having a minimum inhibitory concentration values between 4 and 64 μM [40]. The study mined the proteome of nine species, reducing the number of peptides from 63,343 to just 11 that were selected for in vitro validation, highlighting the ability of AI to navigate vast proteomic datasets effectively.

In a more advanced approach, more than one million novel AMPs from the global microbiome were identified by analyzing over 63,000 metagenomes and 87,000 prokaryotic genomes [41]. This was achieved using the Macrel machine learning pipeline, which is based on a random forest algorithm. After validation, among the 100 synthesized peptides, 79 showed antimicrobial activity, ensuring the accurate predictive performance of the method [41].

Pushing the boundaries further, a multitasking deep learning model like APEX was designed to systematically mine the complete proteomes of extinct organisms [42]. It employs an encoder neural network, using both recurrent and attention neural networks, coupled with fully connected neural networks, for regression and classification. By assembling predictions from 40 independently trained models, APEX outperforms random forest, gradient boosting, and elastic net ML methods. For the dataset, both in-house and external datasets were combined, and multitask constraints were effective at maximizing generalizability [42]. By mining over 10 million peptide fragments from the proteomes of 208 extinct species, they found 37,176 antimicrobial peptides with broad spectrum, offering a novel chemical space for AMP discovery.

More recently, Li et al. (2025) identified 8008 novel AMPs by mining UniProtKB/Swiss-Prot using two AMPlify models: imbalanced and balanced [43]. The imbalanced model handles large, highly imbalanced datasets with many inactive AMPs, while the balanced model works best on smaller, high-confidence datasets [43]. By applying both models, they select only sequences predicted by both as AMPs, thus reducing false positives [43]. This dual-model strategy was successful for mining large protein databases with many non-AMPs.

Artificial intelligence (AI) is transforming proteome mining and dataset development by paving the way for effective processing, pattern recognition, and the prediction of highly complex, high-dimensional proteomics data. AI-based methods have accelerated the discovery of novel antimicrobial peptides (AMPs), even uncovering candidates from extinct organisms and underexplored habitats, offering an untapped source of antimicrobials. Though significant challenges exist, the majority of current deep learning models are based only on sequence data, overlooking structural information that could improve predictive accuracy. Furthermore, combining proteomics with other omics data for comprehensive insight is computationally intensive, and technical limitations like differentiating true coding sequences from false positives persist. The field is further limited by annotation gaps, validation bottlenecks and uneven database coverage. Future research should address these issues by integrating structural and three-dimensional descriptors, refining sampling strategies, and expanding validation across a variety of strains and conditions. This will improve both the scope and accuracy of AI-powered proteomic mining.

#### 3.4.2. AI in AMP Prediction

Several efforts have been made to develop and train classifiers to distinguish between antimicrobial peptides and non-antimicrobial peptides. Unlike small molecule antibiotic classification, which is based on graph-based molecular data, these peptide classification models are based on sequence features. A hierarchical classifier that not only predicts if a peptide is antimicrobial or not, but also categorizes AMPs according to their activity against *Staphylococcus aureus*, was developed [44]. This multi-level classification approach operates sequentially, where the output of one classifier serves as the input for the next. The first classification model was trained using Support Vector Classifier (SVC), Random Forest (RF), and K-Nearest Neighbors (KNN), achieving high accuracy and an F1-score above 0.9 for classifying AMPs and non-AMPs. For the second classifier, the random forest model demonstrated a better performance compared to linear SVC and KNN models [44]. This approach demonstrates the value of integrating two classifiers to enable strain-specific AMP discovery.

Building on the strengths of transformer-based architectures, advanced models have been developed using the BERT model coupled with a multilayer perceptron [45]. This model outperforms others and achieves an AUC of 0.962 on an independent test set, facilitating the identification of promising candidates, such as peptide A-222, which shows broad-spectrum activity against both Gram-positive and Gram-negative bacteria [45].

As another method for AMPs discovery, the ABPCaps model is designed specifically to use a capsule network-based framework to identify antibacterial peptides (ABP) [46]. Unlike other neural networks, capsule networks employ vector-valued units that learn hierarchical relationships and encode both the presence and detailed properties of sequence patterns [46]. ABPCaps overcomes the limitations of CNNs and RNNs, like loss of spatial information and pooling invariance, by combining 1D convolution for local feature extraction, LSTM layers for capturing long-range dependencies, and capsule layers for hierarchical representation. This model achieved an accuracy of 93.33%, an F1-score of 0.9134, and an AUROC of 0.9289, outperforming LSTM, GRU, CNN, KNN, and RF [46].

Finally, the discovery of the potent antibacterial PA-Win2 shows the value of multi-task deep learning [47]. The authors used a deep multi-task learning model based on bidirectional long short-term memory (BiLSTM) to identify this peptide with low MIC against *Bacillus subtilis*, *Pseudomonas aeruginosa*, and multidrug-resistant *P. aeruginosa* [47]. This multi-task learning strategy improves generalization, overcomes data scarcity, and shows better performance than single-task models.

#### 3.4.3. AMPs Prediction Softwares

Instead of focusing on developing new models for AMP prediction, efforts have also been dedicated to creating user-friendly software tools. These publicly available platforms are developed to facilitate the prediction of antimicrobial peptides, making advanced computational approaches accessible to researchers without extensive programming expertise.

For instance, Lin et al. 2021 developed the AI4AMP, by encoding AMPs through a hybrid CNN-LSTM. The encoding considered physicochemical component 6 (PC6), which captures six key physicochemical properties for each amino acid within a peptide sequence [48]. The AMPlify prediction model was implemented by integrating two attention mechanisms atop a bidirectional LSTM architecture. It processes AMP sequences encoded with positional information, allowing the model to recurrently capture contextual dependencies throughout the input sequence [49].

Similarly, Jan et al. (2022) developed a dynamic and high-throughput classification model using a Support Vector Machine classifier, achieving a high performance with an accuracy of 97.07% [50]. The model’s encoding was based on a hybrid combination of Position-Specific Scoring Matrix (PSSM), dipeptide composition (DPC), and pseudo amino acid composition (PseAAC) features to extract sequence and evolutionary information. However, the predictor is not yet launched for public use. Furthermore, Ruiz Puentes et al. (2022) developed an innovative strategy for AMPs discovery using Graph convolutional networks, where peptides are represented as graphs, to capture spatial and physicochemical features that are important for enhancing prediction accuracy [51]. When screening *E. coli* genome, AMP-Net showed successful identification capabilities, achieving 95.76% average precision for binary AMP prediction [51]. The study highlights that using graph networks for peptide encoding offers superior predictive performance over traditional sequence-based methods, which consider only the sequence of amino acids and ignore structural interactions.

AMP-BERT is a deep learning model based on a fine-tuned bidirectional encoder representation from the transformer (BERT) architecture. It is specifically designed to capture the structural and functional features of peptide sequences and classify them as AMP or non-AMP [52]. Unlike traditional deep learning models like CNNs or LSTMs, which extract local or long-range patterns of sequential data, BERT employed a transformer attention mechanism and positional encoding to understand the global context across the peptide sequence and make better predictions. The model achieved an F1 score of 0.9278 during validation, confirming that the model can be applied to unseen data after learning with training samples [52].

AMP-RNNpro is a two-stage framework for detecting antimicrobial peptides (AMPs) that is based on a Recurrent Neural Network (RNN) architecture [53]. The novelty of this model is how it represents protein sequences before feeding them to the neural network. It combines eight different methods to encode the amino acid sequences, instead of using just one [53]. These methods are used based on four types of information: amino acid composition, grouped composition, autocorrelation, and pseudo-amino acid composition. In the first stage, the six top-performing models (Random Forest, K-nearest Neighbor, Extreme Gradient Boosting Classifier, Voting Classifier, and Extra-Trees Classifier) extract probabilistic features from eight sequence encodings. In the second stage, these probabilities are merged and serve as input to a final Recurrent Neural Network meta-model. This framework excels in the identification of novel AMPs, achieving 97.15% accuracy, 96.48% sensitivity, and 97.87% specificity [53]. The study highlights that integrating multiple different sequence feature encodings with probabilistic outputs from diverse performing machine learning models, and combining them with a recurrent neural network, results in the highly accurate and balanced identification of antimicrobial peptides.

AMPActiPred, a three-stage prediction framework based on a deep forest architecture designed to identify ABP, predicts their bacterial species target, and estimates their activity level (MIC) [54]. This framework incorporates five peptide descriptors to combine diverse peptide characteristics into ABP prediction: amino acid composition, dipeptide composition, pseudo-amino acid composition, the composition of k-spaced amino acid group pairs, and physicochemical properties. On independent tests, the model achieved 87.6% accuracy, 91% specificity, 82.6% sensitivity for ABP prediction, and strong performance in target prediction [54]. The main AI-based AMP prediction platforms, their architectures, reported performance, and availability are summarized (Table 5).

While most current AMP-focused AI models rely predominantly on sequence-derived features, key mechanistic and biophysical constraint such as membrane disruption mechanisms, peptide aggregation, proteolytic stability, immunogenicity, and off-target cytotoxicity are only partially addressed. These properties strongly influence antimicrobial selectivity and in vivo efficacy but are difficult to infer from sequence data. Integrating biophysical modeling, structural descriptors, and multi-task learning frameworks will be essential to move beyond purely sequence-based prediction toward mechanism-informed AMP design.

#### 3.4.4. AI in AMPs De Novo Generation

Moving beyond prediction, the discovery of new peptides represents a vital step in antimicrobial discovery, as potential lead compounds must exhibit the necessary pharmacological properties to be therapeutically effective. De novo antimicrobial design focus on generating and optimizing novel peptides from scratch. The development mostly depends on learning features from known sequences and generating new ones based on these features. Autoencoders have been widely adopted in de novo peptide design due to their ability to learn compressed, informative latent representations that enable the generation of novel sequences with the desired biological properties.

Variational Autoencoders (VAEs) are among the most adopted models, using LSTM-based encoder and decoder networks to learn latent representations of AMPs [55]. Trained in 3280 AMP sequences and corresponding MIC values against *E. coli*, the model used a loss function that combines reconstruction loss and KL loss to confirm both accurate sequence regeneration and regularization of the latent space. Applying cosine similarity to latent vectors helps direct targeted sampling close to active and inactive peptides and enables the controlled generation of novel sequences [55]. Visualization of the latent space in just two dimensions using PCA (Principal Component Analysis), t-SNE (t-distributed Stochastic Neighbor Embedding), and UMAP (Uniform Manifold Approximation and Projection) reveals activity-based clustering. A Gradient Boosting regression model was used for the prediction of MIC values, which enabled targeted sampling from the latent space to generate 38 new peptides. Experimental validation showed that peptides sampled near highly active regions had lower MICs (as low as 2 μM), confirming the model’s ability to generate biologically active AMPs with predictive accuracy (R^2^ = 0.73, RMSE = 0.50) [55].

Building on these advances, Pandi et al. (2023) introduced an innovative deep learning framework for the design of de novo AMPs using two versions of a VAEs: VAE-v1 without KL-term annealing and VAE-v2 with KL-term annealing [56]. The VAE architecture includes an encoder (five CNN layers), latent space (vector size 50) and a decoder (four deconvolutional layers + GRU layer) [56]. The training process involved pretraining on a dataset of 1.5 million peptide sequences, followed by fine-tuning via transfer learning to specialize the latent space in AMP features using 5319 experimentally validated AMP sequences. Random sampling, neighborhood sampling around known AMPs, and optimization-based sampling strategies were used to generate 500,000 candidate peptides. Then, a CNN and RNN-based regressors were used to predict minimum inhibitory concentrations (MIC) [56]. However, the functional hit rate (6% success rate) can be further improved through better sampling or model refinement or data preprocessing. Beyond conventional VAEs, alternative autoencoder frameworks such as the Wasserstein Autoencoder (WAE) have also demonstrated great potential for de novo peptide design, offering advantages in learning smoother and more structured latent spaces.

For instance, Das et al. (2021) combined Wasserstein Autoencoder (WAE) to learn a latent space from 1.7 million peptide sequences, with a Conditional Latent attribute Space Sampling (CLaSS), a novel sampling method from the latent space for sequence generation [57]. To predict toxicity and antimicrobial activity, a deep neural network including LSTM was applied, and a molecular dynamics simulation was conducted to screen peptide–membrane interactions. Impressively, the entire pipeline, from model training to in vivo testing, was completed in just 48 days. However, only two peptides out of the 20 synthesized were active against *S. aureus*, *E. coli*, *P. aeruginosa*, and MDR *K. pneumoniae* [57]. The 10% success rate indicates that the latent space includes many peptides that are either not stable, toxic, or not antimicrobial. Maybe, instead of using a classifier after generation, guided generation during training using a conditional Generative Adversarial Network (cGAN) could improve results with more focused candidates.

Generative Adversial Networks (GANs) have also gained attention in the generation of novel AMPs. Modified strategies, such as Wasserstein GAN with gradient penalty (WGAN-GP), helped improve training stability and prevent mode collapse by gradient penalty, addressing common challenges in original GAN frameworks. The evaluation of the generated peptides showed that their amino acid compositions and physicochemical properties are very similar to real AMPs, and the t-SNE visualization confirmed they are clustered near AMPs in the reduced feature space, indicating that the WGAN-GP model effectively learned meaningful and relevant biological patterns [58].

Explainable AI has emerged as a complementary direction, combining both rough set theory, a rule-based ML method for AMP classification, with a codon-based genetic algorithm (CB-GA) for sequence generation and optimization [59]. The Rough Set Theory (CLN-MLEM2), which separates active AMPs from inactive ones, has shown high-specificity performance when using AAindex1 amino acid descriptors to generate interpretable rule sets, highlighting the benefit of integrating explainable AI in antimicrobial compounds’ discovery [59]. This approach allows researchers to understand why certain sequences are classified as AMPs, unlike many “black-box” deep learning models, where the decisions remain opaque. On the other hand, CB-GA converts known peptide sequences to codon-based DNA representation, enabling the generation of novel sequences through mutation, crossover, and codon variability [59]. However, only three peptides were synthesized, with only one showing activity against *S. epidermis*, which limits the generalizability of the model’s predictive performance.

More recently, an explainable deep learning framework for AMPs prediction and optimization has been developed, fusing four deep learning architectures (CNN, LSTM, Attention, Transformer) into the ensemble AMP-CLIP classifier (precision of 0.99, accuracy of 0.874) and AMP-READ for the regression of antimicrobial potency (MIC) [60]. To solve the ‘black box’ limitation of AMP-READ, they developed EvoGradient, a virtual directed evolution algorithm that uses model-derived gradients to identify and optimize key amino acids, generating potent AMP variants and improving model explainability [60]. They successfully achieved the virtual evolution of 32 peptides into potent AMPs, demonstrating the importance of integrating explainable AI into drug discovery.

Other innovative methodologies have emerged in this area; the database Filtering Technology is one such approach, which facilitates the design of new peptides by identifying and utilizing the most probable parameters shared among a group of peptides exhibiting similar biological activity. Bobde et al. (2021) used this technique with positional frequency analysis to design eight novel synthetic antimicrobial peptides (PHNX-1 to PHNX-8) targeting Gram-negative bacteria [61]. Then, several machine learning classifiers were used to predict their antimicrobial potential; PHNX-1 was the best-performing peptide, with an MIC value between 16 and 64 μg/mL against *E. coli*.

Zervou et al. (2024) furthered this by presenting a complementary approach based on Feedback Generative Adversarial Networks (FBGAN) for AMP design, in which generation is guided by classifier feedback rather than a latent sampling space [62]. By integrating two enhanced classifiers—one using k-mers-based encoding and the other using ESM2 protein language model embeddings—the FBGAN-ESM2 achieved superior performance in both AMP prediction accuracy and the physicochemical value of generated peptides when compared to state-of-the-art models like AMPGAN and HydrAMP [62]. Cao et al. (2024) proposed a three-stage pre-trained framework, TG-CDDPM, for generating diverse AMPs using a text-guided conditional denoising diffusion probabilistic model (DDPM) [63]. In contrast to traditional LSTM-, VAE-, or GAN-based methods which generate peptides by sampling from latent space with limited controllability, TG-CDDPM incorporates peptide attribute information in a text-guidance manner [63]. Using large-scale datasets with paired peptide sequences and textual descriptions, the authors combined contrastive learning for text–peptide alignment, an adapter for improved conditional inference, and a transformer-based diffusion model for sequence generation. Compared to other methods, TG-CDDPM achieved the highest AMscores for predicted antimicrobial activity, and the generated peptides show good physicochemical features and strong membrane penetration in molecular dynamics simulations [63].

Relying only on textual guidance and lacking control over secondary structure limits the model’s efficacy, as the secondary structure is a key aspect for functional AMPs. Future work should integrate structural and functional attribute guidance into the generation process. An integrated summary of the models employed for AMP discovery, including predictive, generative, and explainable AI frameworks, is presented in Table 6.

## 4. Discussion

The average human lifespan has increased due to antibiotics. However, decades of inappropriate use have fueled antimicrobial resistance (AMR) [2], with multidrug-resistant pathogens rendering many current antibiotics ineffective [19]. Scientists have pursued various strategies to discover new antimicrobials, including isolating compounds from previously unculturable microorganisms, mining human and animal microbiomes for bioactive compounds, and using advanced laboratory and computational approaches to screen and optimize candidates [64]. Most AI-driven efforts focus on antimicrobial peptides (AMPs) rather than small molecules due to their broad-spectrum activity, rapid bactericidal action, and limited toxicity [65]. Unlike small molecules, bacteria develop resistance to AMPs more slowly, as they disrupt membranes and interfere with multiple intracellular targets [65]. Computational technologies have consistently shaped antimicrobial discovery. This review provides an overview of recent methodologies, computational strategies, and AI-driven models employed for prediction or de novo generation, including both machine learning classifiers and deep learning models.

Most studies focused on predictive AI models for virtual screening, for example, graph neural networks like Directed Message Passing Neural Networks (D-MPNNs) achieved high accuracy (ROC-AUC 0.8–0.9) in classifying compounds as active or inactive. This model identified novel compounds such as halicin with broad-spectrum antimicrobial activity and abaucin with narrow-spectrum activity against *A. baumannii* [30,31]. The molecular design via attribute-guided search (MDAGS) model reflects the convergence of predictive and generative approaches by combining a graph-based encoder–predictor and a transformer-based decoder generator. This approach improved the model’s ability to produce chemically valid and novel structures, as reflected in its outstanding performance on generative metrics: with a validity 95.6%, novelty 99.3%, and uniqueness 99.8% [36].

While broad-spectrum antibiotics enhance selection and the emergence of resistant strains, focusing on their development may not be effective in addressing antimicrobial resistance. Instead, the discovery of narrow-spectrum, species-specific antimicrobials that target certain diseases is required. With the aid of artificial intelligence, it is now possible to recognize unique bacterial components, which allow for the design of precisely targeted drugs to treat specific infections. The strategy introduced by Khabaz et al. exemplifies this paradigm shift: their hierarchical machine learning model is specifically trained to predict antimicrobial peptide activity against *Staphylococcus aureus*, instead of depending on broad AMP/non-AMP classification [44]. By combining feature selection and species-specific training, their approach shows how AI-driven models can facilitate the discovery of narrow-spectrum, strain-specific antimicrobials. Such targeted approaches not only improve therapeutic precision but also help minimize collateral damage to the microbiome and reduce the risk of the development of resistance.

Recent advances in AI-driven proteomic and genomic data mining have accelerated AMP discovery. The design of the computational workflow still has a significant impact on the quality and interpretability of the results. Studies using hybrid workflows that incorporate physicochemical pre-filters with conventional classifiers show that a simple rule-based screening can produce biologically meaningful candidates with relatively small datasets [40]. However, these approaches are still limited by their dependency on predefined features and the risk of ignoring unconventional AMP scaffolds. In contrast, large-scale mining frameworks like the Macrel random forest pipeline, and multitask deep learning ensembles like APEX, show the scalability of AI, but also highlight critical problems with overfitting, biological validation bottlenecks, and redundancy control. While the Macrel pipeline’s 79% in vitro confirmation rate indicates strong generalization to a variety of microbiomes, its reliance on imbalanced, noisy metagenomic data and short-sequence alignments raises questions about false-negative bias and the reproducibility of hits across species [41].

In AMP prediction, deep learning models clearly outperform traditional feature-based classifiers, yet their advantages extend beyond accuracy metrics, as they can capture meaningful hierarchical biological patterns. Transformer and attention-based architectures dominate this space because they can contextualize amino acid relationships across long sequences, which is a critical property for functional specificity. For instance, the Bidirectional Long Short-Term Memory (BiLSTM) attention model AMPlify achieved 93.7% accuracy on benchmark datasets, but its strength lies less in raw performance and more in interpretability: the attention layer highlights the residues that contribute most to antimicrobial activity, offering a biological insight absent in earlier models [49]. Optimized BERT-based models show high performance in capturing global sequence context and achieved the best outcomes of all tested methods [52].

Generative AI models can generate novel AMP candidates with the required features rather than classifying existing candidates. Variational autoencoders and Wasserstein autoencoders have shown that latent-space representations can produce chemically plausible and functionally diverse sequences; their low hit rates (≈5–10%) highlight the susceptibility of generation when trained on limited or noisy data [56,57]. Newer frameworks, such as the text-guided conditional denoising diffusion probabilistic model, outperform previous VAE and GAN models on AMP-likeness metrics, and the generated candidates show strong membrane-penetration in molecular dynamics simulations [63].

A notable gap is the lack of repetitive experimental feedback; none of the AI frameworks reported in this review integrate a loop where the in vitro results iteratively guide and refine the model’s predictions. Another common limitation is the focus on the optimization of antimicrobial activity without optimizing the pharmacological profiles. In silico models based on only antimicrobial activity can end up with candidates having poor pharmacokinetic or high toxicity properties. The lack of explainability is another challenge, deep neural networks work as “black boxes”, making it difficult to understand why given molecules or peptides were predicted or generated to be active. Data-related limitations were reported by most of the studies. AI models generally need large, high-quality training sets, but the available antimicrobial data are often scarce, unbalanced, and dominated by certain bacterial strains or chemical classes [25]. Models trained on limited or biased data lead to the rediscovery of known or invalid scaffolds. Dataset quality and annotation consistency often exert a greater influence on downstream performance than the model architecture itself, underscoring data curation as a primary bottleneck in the field.

Despite the substantial progress in AI-assisted antimicrobial discovery, its translation into clinically viable products remains constrained by well-recognized industrial and biological bottlenecks. A major driver of late-stage failure is ADMET attrition, as many AI-prioritized compounds are optimized for target activity or in silico metrics while overlooking the absorption, metabolic stability, off-target toxicity, and pharmacokinetic limitations that emerge during experimental validation. Cost and scale-up further limit applicability, since the synthetically complex or low-yield molecules generated by AI models are often incompatible with economically viable manufacturing pipelines, particularly under GMP conditions—a challenge that is especially acute in the antimicrobial sector. For peptide-based strategies, including AI-designed antimicrobial peptides (AMPs), immunogenicity remains a significant translational barrier, as sequence novelty and structural instability can provoke host immune responses, rapid clearance, or reduced in vivo efficacy, which are rarely captured by current generative models. In parallel, toxicity prediction failures persist: although AI-based classifiers are widely used, they frequently rely on biased or incomplete datasets and exhibit limited generalizability, leading to false negatives that are revealed only during preclinical testing. Collectively, these issues underpin the high attrition rates of AI-enabled drug discovery and underscore the gap between computational success and clinical feasibility, highlighting the need for the closer integration of AI with experimental pharmacology, early developability assessments, and realistic industrial constraints [66,67,68].

As a final comment, we should always be aware of AI results. Many generative AI outputs remain chemically unstable, synthetically inaccessible, or biologically irrelevant without extensive expert curation. We therefore caution against extrapolating in silico success to clinical feasibility.

### Future Perspectives

Several future aspects can overcome the mentioned limitations and improve antimicrobial discovery. One potential direction is the development of 3D structure-aware generative models. While most models use 2D molecular representations, graphs, or SMILES strings, the effectiveness of an antibiotic often relies on 3D conformations and target interactions. Integrating spatial molecular properties and protein structure can enable structure-based generation using 3D equivariant graph neural networks that preserve translational molecular symmetries [69]. These models could generate candidates with good binding affinity to a target’s 3D pocket and a favorable orientation and dynamics, potentially leading to new mechanisms of action. AlphaFold2 can predict 3D protein structures to near experimental accuracy in most cases [70]. A GNN classifier (deepAMPNet) trained using AlphaFold2-predicted AMP structures illustrates how 3D models can enhance the identification and design of novel active peptides [71].

Together, active learning approaches and reinforcement learning (RL) provide opportunities for incorporating guided feedback into the generative process. Closed-loop active learning platforms have the potential to revolutionize antibiotic discovery by improving candidate selection through experiment-in-the-loop strategies. For example, the BATCHIE tool, which is a Bayesian sequential design framework, has already demonstrated impressive efficacy in large-scale drug screening by dynamically choosing the most instructive experiments based on prior findings [72]. Future antibiotic pipelines could follow this strategy; generative models propose new candidates, which would then be tested through automated high-throughput assays, and the results would be used to retrain the model.

Another promising avenue is to enhance the explainability and transparency of models. Graph attribute methods and attention mechanisms can be integrated into model design to address why a given prediction was made [73]. These techniques can reveal which AMP motif or pharmacophore drives the predicted activity.

Future frameworks should better incorporate pharmacokinetic and toxicity considerations and model the potential evolution of resistance. As discussed above, current AI models rarely optimize multiple objectives, such as potency and low toxicity, simultaneously, underscoring the need for improved multi-objective optimization strategies. Reinforcement learning represents a promising approach by penalizing generated molecules that violate drug-likeness, toxicity, or developability constraints. Moreover, the integration of structural biology, molecular simulation, multi-omic data, and resistance evolution modeling is expected to improve mechanistic resolution, clinical relevance, and validation fidelity in AI-driven antimicrobial discovery. Close collaboration between computational scientists and experimental biologists will therefore be essential. This review underscores that algorithms alone cannot solve antibiotic discovery and must be integrated through robust experimental validation and synthesis efforts.

## 5. Conclusions

Artificial intelligence has rapidly emerged as a transformative force in antimicrobial discovery, offering powerful solutions to long-standing challenges associated with antimicrobial resistance. By enabling the rapid analysis of large and complex datasets, AI-driven models have expanded the accessible chemical and biological space, accelerated candidate prioritization, and facilitated the design of structurally novel small-molecule antibiotics and antimicrobial peptides. As highlighted throughout this review, advances in graph-based learning, attention-driven architectures, and generative frameworks have collectively reshaped how antimicrobial candidates are identified and optimized. Beyond performance improvements, a notable conceptual shift is underway toward precision-oriented antimicrobial strategies. AI methodologies increasingly support the discovery of narrow-spectrum agents and target-specific antimicrobial peptides, reflecting a growing emphasis on minimizing resistance selection pressure and preserving microbiome integrity. In parallel, explainable and interpretable AI models are gaining prominence, enabling deeper biological insight and fostering greater confidence in computational predictions. Despite these advances, substantial barriers remain before AI-enabled antimicrobial discovery can be fully translated into clinical practice. Persistent challenges include the limited availability of high-quality, balanced, and experimentally annotated datasets; the insufficient integration of pharmacokinetic, toxicity, and resistance-evolution considerations; and a continued gap between in silico predictions and robust experimental validation. Addressing these limitations will require tighter coupling between computational modeling and experimental feedback, as well as the broader adoption of multimodal and structure-aware learning frameworks. Looking forward, the next generation of AI-driven antimicrobial discovery is likely to be defined by hybrid pipelines that integrate predictive modeling, generative design, and experimental validation in iterative loops. The incorporation of three-dimensional structural information, multi-omic data, and clinically relevant endpoints will be critical for enhancing translational reliability. Ultimately, interdisciplinary collaboration between microbiologists, chemists, data scientists, and clinicians will be essential to ensure that AI evolves from a powerful exploratory tool into a reliable engine for delivering next-generation antimicrobial therapies.

## 6. Declaration of Generative AI and AI-Assisted Technologies in the Writing Process

During the preparation of this work, the authors used ChatGPT-5.2 in order to correct and improve the readability and language of the manuscript. After using this tool, the authors reviewed and edited the content as needed and take full responsibility for the content of the published article.

## Figures and Tables

**Table 1:** Query strings used for information retrieval across databases.

Database	Search Strings
PubMed	((((‘antimicrobial resistance’ [Title/Abstract] OR ‘resistant bacteria’ [Title/Abstract] OR ’Resistant microorganisms’ [Title/Abstract])) AND ((‘artificial intelligence’ [Title/Abstract] OR ‘deep learning’ [Title/Abstract] OR ‘machine learning’ [Title/Abstract] OR ‘generative AI’ [Title/Abstract] OR ‘explainable AI’ [Title/Abstract] OR ‘predictive AI’ [Title/Abstract]))) AND ((‘antibiotic discovery’ [Title/Abstract] OR ‘drug discovery’ [Title/Abstract] OR ’novel drug’ [Title/Abstract])))
Scopus	TITLE-ABS-KEY (“antimicrobial resistance” OR “resistant bacteria” OR “resistant microorganisms”) AND (“artificial intelligence” OR “deep learning” OR “machine learning” OR “generative AI”) AND (“antibiotic discovery” OR “drug discovery” OR “novel drug”) AND PUBYEAR > 2019 AND PUBYEAR < 2026
Web of Science	((‘antimicrobial resistance’ OR ‘resistant bacteria’ OR ‘resistant microorganisms’) AND (‘artificial intelligence’ OR ‘deep learning’ OR ‘machine learning’ OR ‘generative AI’ OR ‘explainable AI’ OR ‘predictive AI’) AND (‘antibiotic discovery’ OR ‘drug discovery’ OR ‘novel drug’))

**Table 2:** Characteristics and key findings of the included studies on predictive models for SMAs discovery.

Paper	Objective	Model	Dataset	Performance Metrics	Key Results
Stokes et al. (2020) [30]	Screen millions of molecules from large chemical libraries to identify new, structurally unique antibiotics effective against priority pathogens and resistant strains.	Directed–Message Passing Neural Network (D-MPNN)	Training: 2335 molecules (FDA-approved drug and natural product). Screening: 107 million molecules (ZINC15)	ROC-AUC = 0.896	Halicin, a novel molecule with broad-spectrum activity against multidrug-resistant *E. coli*, tuberculosis, and carbapenem-resistant Enterobacteriaceae in vitro; treated *A. baumannii* infections in mouse models.
Rahman et al. (2022) [32]	Screen small-molecule libraries to identify antibacterial compounds against *Burkholderia cenocepacia*.	D-MPNN	Training: 29,537 molecules. Screening: 225,819 (FDA-approved drug and natural product)	ROC-AUC = 0.823; PRC-AUC = 0.241; F1 = 0.104; MCC = 0.167	21 FDA-predicted compounds and 5 natural products were active; two unreported antibacterials (STL558147, PHAR261659) showed broad activity. Limited chemical diversity and class imbalance constrained model generalization.
Liu et al. (2023) [31]	Discover structurally and functionally new molecules with activity against *A. baumannii*.	D-MPNN	Training: 7684 small molecules. Screening: 6680 molecules	ROC-AUC = 0.792; PR-AUC = 0.337	Abaucin identified with narrow-spectrum antibacterial activity against *A. baumannii*.
Boulaamane et al. (2024) [33]	Screened a large library of natural products with potential activity against *A. baumannii*, focusing on OmpW.	CNN; SVM; Random Forest; k-NN; Gaussian Naïve Bayes	Training: 3196 bioactive compounds. Screening: 11,648 natural compounds	AUC (CNN) = 0.96	Desmethoxycurcumin active against all *A. baumannii* strains in monotherapy and with colistin.
Wang et al. (2024) [34]	Develop a workflow combining machine learning and a combinatorial library to accelerate screening of antibacterials against MRSA.	UMAP + Latent Space Constraint Neural Network (LSCNN)	Full combinatorial library: 111,720 compounds. Initial training set: 360 synthesized	R≈0.94; RMSE≈0.09; Hit rate = 60%	Compounds H4–H6 showed excellent activity (MIC = 12 µM) against MRSA with reduced resistance development.
Olayo-Alarcón et al. (2025) [35]	Construct a lightweight, data-efficient predictive framework (MolE) that prioritizes novel antimicrobial compounds using self-supervised molecular representations.	MolE and XGBoost	Pre-training: 100,000 unlabeled molecules. Testing: 100,000 molecules. Screening: 2320 molecules	ROC-AUC ≈ 92.85%	Discovered and confirmed three non-antibiotic drugs as effective inhibitors of *Staphylococcus aureus*; capable of predicting broad-spectrum antibiotics.

**Table 3:** Characteristics and key findings of included studies on SMAs de novo design.

Paper	Objective	Model	Dataset	Performance Metrics	Key Results
Chen et al. (2023) [36]	Develop a generative approach combining attribute prediction models with an efficient guided search strategy to design potent antibiotic molecules exhibiting desired antibacterial activity.	Encoder–predictor (D-MPNN), Generator (GPT-based)	Main: 2334 molecules (FDA-approved + natural products). Pretraining: MOSES (1.6 M train), GuacaMol (1.3 M train).	Validity = 0.956; Novelty = 0.993; Uniqueness = 0.998; FCD = 0.874	MDAGS generated novel molecules with better predicted antibacterial activity than existing antibiotics while preserving similarity to functional analogs. Limitations include lack of toxicity and PK considerations.
Krishnan et al. (2023) [37]	Generate novel anti-tuberculosis candidates targeting the Mtb chorismate mutase protein using a structure-based de novo design algorithm.	GAT-VAE, SMILES-VAE, DTA model	Training: 5981 binding site graphs; 1.6M drug-like molecules.	SMILES-VAE: Acc = 93.22%, Novelty = 96%; GAT-VAE: ROC = 0.89	4041 binding-compatible molecules generated; 75% showed high pharmacophore similarity. Limitation: no in vitro validation.

**Table 4:** AI Models for Small Molecule Antibiotic Discovery.

Task	Model	Strength	Limitations
Prediction, classification	D-MPNN [30]	Captures molecular representations directly from graph structure, uses directed messages.	Limited ability to extract molecule-level representations during message passing for large complex molecules.
	CNN [33]	Extracts complex, non-linear relationships and patterns from molecular descriptors, molecular graphs/images.	Can overfit small or noisy datasets.
	LSCNN [34]	Imposing constraints (Euclidean or contrastive loss) in hidden layers stabilizes training, improves prediction accuracy.	Requires careful design of latent space constraints.
	XGBoost [35]	Employs regularization (L1, L2 penalties), tree pruning, and built-in cross-validation to reduce overfitting.	Performance depends on the quality and relevance of input features (MolE, ECFP4, chemical descriptors).
De novo generation	GPT [36]	Generates novel antibiotic candidates with high structural diversity.	Lacks explicit knowledge of chemical constraints (valence, charge, stereochemistry).
	GAT-VAE [37]	Captures complex interactions between amino acid residues of ligand’s binding sites.	Requires large, diverse, high-quality datasets.
	SMILES-VAE [37]	Effective for learning chemical syntax directly from SMILES.	Can generate invalid SMILES.

**Table 5:** AI-based tools and performance metrics for antimicrobial peptide (AMP) prediction.

Software	Metrics	Link
AI4AMP [48] (2021)	Acc: 0.8850, Pre: 0.9035, Sen: 0.8620, Spe: 0.9080, F1 score: 0.8822, MCC: 0.7707	http://symbiosis.iis.sinica.edu.tw/PC_6/
AMPlify [49] (2022)	Acc: 93.71%, Sen: 92.93%, Spe: 94.49%, F1 score: 93.66%, AUROC: 98.37%	https://github.com/bcgsc/AMPlify
Target-AMP [50] (2022)	Acc: 97.07%, Sen: 91.68%, Spe: 98.79%, Pre: 93.82%, MCC: 0.91	https://ars.els-cdn.com/content/image/1-s2.0-S0003269713000390-mmc1.pdf https://ars.els-cdn.com/content/image/1-s2.0-S0003269713000390-mmc2.pdf https://ars.els-cdn.com/content/image/1-s2.0-S0003269713000390-mmc3.pdf
AMPs-Net [51] (2022)	Acc: 89.81%, Pre: 95.76%	https://github.com/BCV-Uniandes/AMPs-Net
AMP-BERT [52] (2023)	Acc: 0.9280, AUROC: 0.9665, AUPR: 0.9653, Sen: 0.9262, Spe: 0.9303, F1 score: 0.9278	https://github.com/GIST-CSBL/AMP-BERT
AMP-RNNpro [53] (2024)	Acc: 97.15%, Sen: 96.48%, Spe: 97.87%	https://github.com/Shazzad-Shaon3404/Antimicrobials_
AMPActiPred [54] (2024)	Acc: 0.876, Spe: 0.910, Sen: 0.826, MCC: 0.742	https://github.com/lantianyao/AMPActiPred

Programs listed in this table were accessed during manuscript preparation (July–December 2025).

**Table 6:** Characteristics and key findings of included studies on AI models for AMP discovery.

Paper	Objective	Model/Framework	Dataset	Performance Metrics/Key Results
Duque Salazar et al. (2020) [40]	Identify novel AMPs from nine organisms’ proteomes	PepMultiFinder algorithm, CAMPr3	63,343 proteins of nine species from UniProt	10/11 peptides showed antimicrobial activity (MIC = 4–64 µM), Coco2: Best candidate (MIC = 4 µM vs. *P. aeruginosa*, no hemolysis, no cytotoxicity)
Boone et al. (2021) [59]	Design novel AMPs against S. epidermidis using transparent ML + genetic algorithms	Codon-Based GA (CB-GA) + Rough Set Theory (CLN-MLEM2)	Positive: 1274 AMPs, Negative: 1440 non-AMPs from iAMP-2L	Aggregation score = −0.02; AMP-2 showed strong activity (inhibition zone = 1.2 cm at 4 mg/mL); CB-GA enabled wider sequence diversity
Bobde et al. (2021) [61]	Design new AMPs against Gram-negative using ab initio	Database Filtering Technology (DFT) + Positional Analysis + ML predictors	APD3: 594 AMPs + 299 AMPs	Designed 8 PHNX peptides; PHNX-1 showed broad-spectrum activity
Dean et al. (2021) [55]	PepVAE: peptide-generation framework with AMP predictors	Variational Autoencoder + LSTM + regression-based MIC predictors (XGBoost, LightGBM, RF, GB)	GRAMPA: 6760 AMP sequences + 51,345 MIC values	t-SNE ARI = 0.62, AMI = 0.59; GB: R^2^ = 0.73, RMSE = 0.50; generated 38 novel AMPs; 6 experimentally validated
Das et al. (2021) [57]	Deep generative + physics-driven simulations for AMP design	Wasserstein Autoencoder, CLaSS, sequence-level LSTM	1.7 M unlabelled + 9000 labelled peptides	AMP classification: WAE = 87.4%, LSTM = 88%; generated 90,000 candidates → 20 synthesized → 2 novel AMPs highly potent
Lin et al. (2021) [48]	Deep learning AMP predictor using PC6 physicochemical encoding	AI4AMP + Deep Learning	Training: 706 sequences; External test: 1130 sequences	Accuracy = 88.5%; precision = 90.35%; sensitivity = 86.2%; specificity = 90.8%; F1 = 88.2%; MCC = 0.77; outperformed previous predictors
Li et al. (2022) [49]	Discover novel AMPs targeting WHO-priority pathogens	Bi-LSTM + MHSDPA + Context Attention	3061 AMPs from APD3, 1923 AMPs from DADP, 4173 non-AMPs	Accuracy = 93.71%; sensitivity = 92.93%; specificity = 94.49%; F1 = 93.66%; AUROC = 98.37%; predicted 16 top candidates—11 synthesized and 4 highly active
Jan et al. (2022) [50]	Efficient classification framework for AMP prediction	KNN, RF, SVM	Training: 3175 peptides; Independent: 1840 peptides	Accuracy = 97.07%; evolutionary + compositional features outperformed single descriptors
Ruiz Puentes et al. (2022) [51]	Deep learning to classify AMPs by functionality	Graph Convolutional Neural Network	23,967 sequences (13,468 AMPs, 10,499 non-AMPs)	Average precision = 95.76%; accuracy = 89.81%. Discovered 2 novel AMPs and 2 novel motifs
Lin et al. (2023) [58]	Generate novel AMPs using deep convolutional GAN	Wasserstein GAN + gradient penalty	3195 AMPs	Seven of eight active candidates; GAN-pep3 lowest MICs, potent vs. resistant strains
Lee et al. (2023) [52]	AMP classification via transformer architecture	NLP-based deep network, BERT	Fine-tuning: 1778 AMPs + 1778 non-AMPs; External test: 2065 AMPs + 1908 non-AMPs	ACC ≈76%; F1 ≈0.792; sensitivity = 87.6%; specificity = 63.52%; AUROC = 0.818. Outperforms prior AMP predictors
Pandi et al. (2023) [56]	Integrated deep learning + cell-free synthesis for AMP design	Deep generative VAE, CNN, RNN	Pretraining: 1,552,476 sequences; 5319 AMPs, 10,612 non-AMPs	12.6% hit rate; 30 de novo AMPs designed, 6 broad-spectrum

## Data Availability

No new data were created or analyzed in this study. Data sharing is not applicable to this article.

## References

[1] Hoyert D.L., Kochanek K.D., Murphy S.L. (1999). Deaths: Final data for 1997. Natl. Vital Stat. Rep..

[2] Aminov R.I. (2010). A brief history of the antibiotic era: Lessons learned and challenges for the future. Front. Microbiol..

[3] Hata S., Ehrlich P., Hata S. (1910). Experimentelle Grundlage der Chemotherapie der Spirillosen. Die Experimentelle Chemotherapie der Spirillosen: (Syphilis, Rückfallfieber, Hühnerspirillose, Frambösie).

[4] Fleming A. (2001). On the antibacterial action of cultures of a penicillium, with special reference to their use in the isolation of *B. influenzae*. Bull. World Health Organ..

[5] Mahajan G.B., Balachandran L. (2012). Antibacterial agents from Actinomycetes—A Review. Front. Biosci..

[6] Bentley R., Bennett J.W. (2003). What Is an Antibiotic? Revisited. Advances in Applied Microbiology.

[7] Ishak A., Mazonakis N., Spernovasilis N., Akinosoglou K., Tsioutis C. (2024). Bactericidal versus bacteriostatic antibacterials: Clinical significance, differences and synergistic potential in clinical practice. J. Antimicrob. Chemother..

[8] Cho H., Uehara T., Bernhardt T.G. (2014). Beta-lactam antibiotics induce a lethal malfunctioning of the bacterial cell wall synthesis machinery. Cell.

[9] Andrade F.F., Silva D., Rodrigues A., Pina-Vaz C. (2020). Colistin update on its mechanism of action and resistance, present and future challenges. Microorganisms.

[10] Bush N.G., Diez-Santos I., Abbott L.R., Maxwell A. (2020). Quinolones: Mechanism, Lethality and Their Contributions to Antibiotic Resistance. Molecules.

[11] Jana S., Deb J.K. (2006). Molecular understanding of aminoglycoside action and resistance. Appl. Microbiol. Biotechnol..

[12] Sköld O. (2000). Sulfonamide resistance: Mechanisms and trends. Drug Resist. Updat..

[13] Davies J., Davies D. (2010). Origins and evolution of antibiotic resistance. Microbiol. Mol. Biol. Rev..

[14] Samreen A.I., Malak H.A., Abulreesh H.H. (2021). Environmental antimicrobial resistance and its drivers: A potential threat to public health. J. Glob. Antimicrob. Resist..

[15] Horton J.S., Taylor T.B. (2023). Mutation bias and adaptation in bacteria. Microbiology.

[16] Kolář M., Urbánek K., Látal T. (2001). Antibiotic selective pressure and development of bacterial resistance. Int. J. Antimicrob. Agents.

[17] Bello-López J.M., Cabrero-Martínez O.A., Ibáñez-Cervantes G., Hernández-Cortez C., Pelcastre-Rodríguez L.I., Gonzalez-Avila L.U., Castro-Escarpulli G. (2019). Horizontal Gene Transfer and Its Association with Antibiotic Resistance in the Genus *Aeromonas* spp. Microorganisms.

[18] Darby E.M., Trampari E., Siasat P., Gaya M.S., Alav I., Webber M.A., Blair J.M.A. (2023). Molecular mechanisms of antibiotic resistance revisited. Nat. Rev. Microbiol..

[19] Miller W.R., Arias C.A. (2024). ESKAPE pathogens: Antimicrobial resistance, epidemiology, clinical impact and therapeutics. Nat. Rev. Microbiol..

[20] GBD 2021 Antimicrobial Resistance Collaborators (2024). Global burden of bacterial antimicrobial resistance 1990–2021: A systematic analysis with forecasts to 2050. Lancet.

[21] Uchil R.R., Kohli G.S., Katekhaye V.M., Swami O.C. (2014). Strategies to combat antimicrobial resistance. J. Clin. Diagn. Res..

[22] de la Fuente-Nunez C., Cesaro A., Hancock R.E.W. (2023). Antibiotic failure: Beyond antimicrobial resistance. Drug Resist. Updat..

[23] DiMasi J.A., Grabowski H.G., Hansen R.W. (2016). Innovation in the pharmaceutical industry: New estimates of R&D costs. J. Health Econ..

[24] Gargate N., Laws M., Rahman K.M. (2025). Current economic and regulatory challenges in developing antibiotics for Gram-negative bacteria. npj Antimicrob. Resist..

[25] Cesaro A., Hoffman S.C., Das P., de la Fuente-Nunez C. (2025). Challenges and applications of artificial intelligence in infectious diseases and antimicrobial resistance. NPJ Antimicrob. Resist..

[26] Rabaan A.A., Alhumaid S., Mutair A.A., Garout M., Abulhamayel Y., Halwani M.A., Alestad J.H., Bshabshe A.A., Sulaiman T., AlFonaisan M.K. (2022). Application of Artificial Intelligence in Combating High Antimicrobial Resistance Rates. Antibiotics.

[27] Ali T., Ahmed S., Aslam M. (2023). Artificial Intelligence for Antimicrobial Resistance Prediction: Challenges and Opportunities towards Practical Implementation. Antibiotics.

[28] David L., Brata A.M., Mogosan C., Pop C., Czako Z., Muresan L., Ismaiel A., Dumitrascu D.I., Leucuta D.C., Stanculete M.F. (2021). Artificial Intelligence and Antibiotic Discovery. Antibiotics.

[29] Ouzzani M., Hammady H., Fedorowicz Z., Elmagarmid A. (2016). Rayyan-a web and mobile app for systematic reviews. Syst. Rev..

[30] Stokes J.M., Yang K., Swanson K., Jin W., Cubillos-Ruiz A., Donghia N.M., MacNair C.R., French S., Carfrae L.A., Bloom-Ackermann Z. (2020). A deep learning approach to antibiotic discovery. Cell.

[31] Liu G., Catacutan D.B., Rathod K., Swanson K., Jin W., Mohammed J.C., Chiappino-Pepe A., Syed S.A., Fragis M., Rachwalski K. (2023). Deep learning-guided discovery of an antibiotic targeting *Acinetobacter baumannii*. Nat. Chem. Biol..

[32] Rahman A.S.M.Z., Liu C., Sturm H., Hogan A.M., Davis R., Hu P., Cardona S.T. (2022). A machine learning model trained on a high-throughput antibacterial screen increases the hit rate of drug discovery. PLoS Comput Biol..

[33] Boulaamane Y., Molina Panadero I., Hmadcha A., Atalaya Rey C., Baammi S., El Allali A., Maurady A., Smani Y. (2024). Antibiotic discovery with artificial intelligence for the treatment of *Acinetobacter baumannii* infections. mSystems.

[34] Wang C., Wu Y., Xue Y., Zou L., Huang Y., Zhang P., Ji J. (2024). Combinatorial discovery of antibacterials via a feature-fusion based machine learning workflow. Chem. Sci..

[35] Olayo-Alarcon R., Amstalden M.K., Zannoni A., Bajramovic M., Sharma C.M., Brochado A.R., Rezaei M., Müller C.L. (2025). Pre-trained molecular representations enable antimicrobial discovery. Nat. Commun..

[36] Chen L., Yu L., Gao L. (2023). Potent antibiotic design via guided search from antibacterial activity evaluations. Bioinformatics.

[37] Krishnan S.R., Bung N., Padhi S., Bulusu G., Misra P., Pal M., Oruganti S., Srinivasan R., Roy A. (2023). De novo design of anti-tuberculosis agents using a structure-based deep learning method. J. Mol. Graph. Model..

[38] Han X., Jia M., Chang Y., Li Y., Wu S. (2022). Directed message passing neural network (D-MPNN) with graph edge attention (GEA) for property prediction of biofuel-relevant species. Energy AI.

[39] Bagal V., Aggarwal R., Vinod P.K., Priyakumar U.D. (2022). MolGPT: Molecular Generation Using a Transformer-Decoder Model. J. Chem. Inf. Model..

[40] Duque-Salazar G., Mendez-Otalvaro E., Ceballos-Arroyo A.M., Orduz S. (2020). Design of antimicrobial and cytolytic peptides by computational analysis of bacterial, algal, and invertebrate proteomes. Amino Acids.

[41] Santos-Júnior C.D., Torres M.D.T., Duan Y., Rodríguez Del Río Á., Schmidt T.S.B., Chong H., Fullam A., Kuhn M., Zhu C., Houseman A. (2024). Discovery of antimicrobial peptides in the global microbiome with machine learning. Cell.

[42] Wan F., Torres M.D.T., Peng J., de la Fuente-Nunez C. (2024). Deep-learning-enabled antibiotic discovery through molecular de-extinction. Nat. Biomed. Eng..

[43] Li C., Sutherland D., Salehi A., Richter A., Lin D., Aninta S.I., Ebrahimikondori H., Yanai A., Coombe L., Warren R.L. (2025). Mining the UniProtKB/Swiss-Prot database for antimicrobial peptides. Protein Sci..

[44] Khabaz H., Rahimi-Nasrabadi M., Keihan A.H. (2023). Hierarchical machine learning model predicts antimicrobial peptide activity against Staphylococcus aureus. Front. Mol. Biosci..

[45] Cao Q., Ge C., Wang X., Harvey P.J., Zhang Z., Ma Y., Wang X., Jia X., Mobli M., Craik D.J. (2023). Designing antimicrobial peptides using deep learning and molecular dynamic simulations. Brief. Bioinform..

[46] Yao L., Pang Y., Wan J., Chung C.-R., Yu J., Guan J., Leung C., Chiang Y.-C., Lee T.-Y. (2023). ABPCaps: A Novel Capsule Network-Based Method for the Prediction of Antibacterial Peptides. Appl. Sci..

[47] Oh J.W., Shin M.K., Park H.R., Kim S., Lee B., Yoo J.S., Chi W.J., Sung J.S. (2024). PA-Win2: In Silico-Based Discovery of a Novel Peptide with Dual Antibacterial and Anti-Biofilm Activity. Antibiotics.

[48] Lin T.-T., Yang L.-Y., Lu I.-H., Cheng W.-C., Hsu Z.-R., Chen S.-H., Lin C.-Y. (2021). AI4AMP: An Antimicrobial Peptide Predictor Using Physicochemical Property-Based Encoding Method and Deep Learning. mSystems.

[49] Li C., Sutherland D., Hammond S.A., Yang C., Taho F., Bergman L., Houston S., Warren R.L., Wong T., Hoang L.M.N. (2022). AMPlify: Attentive deep learning model for discovery of novel antimicrobial peptides effective against WHO priority pathogens. BMC Genom..

[50] Jan A., Hayat M., Wedyan M., Alturki R., Gazzawe F., Ali H., Alarfaj F.K. (2022). Target-AMP: Computational prediction of antimicrobial peptides by coupling sequential information with evolutionary profile. Comput. Biol. Med..

[51] Ruiz Puentes P., Henao M.C., Cifuentes J., Muñoz-Camargo C., Reyes L.H., Cruz J.C., Arbeláez P. (2022). Rational. Rational Discovery of Antimicrobial Peptides by Means of Artificial Intelligence. Membranes.

[52] Lee H., Lee S., Lee I., Nam H.A. (2023). AMP-BERT: Prediction of antimicrobial peptide function based on a BERT model. Protein Sci..

[53] Shaon M.S.H., Karim T., Sultan M.F., Ali M.M., Ahmed K., Hasan M.Z., Moustafa A., Bui F.M., Al-Zahrani F.A. (2024). AMP-RNNpro: A two-stage approach for identification of antimicrobials using probabilistic features. Sci. Rep..

[54] Yao L., Guan J., Xie P., Chung C.R., Deng J., Huang Y., Chiang Y.C., Lee T.Y. (2024). AMPActiPred: A three-stage framework for predicting antibacterial peptides and activity levels with deep forest. Protein Sci..

[55] Dean S.N., Alvarez J.A.E., Zabetakis D., Walper S.A., Malanoski A.P. (2021). PepVAE: Variational Autoencoder Framework for Antimicrobial Peptide Generation and Activity Prediction. Front. Microbiol..

[56] Pandi A., Adam D., Zare A., Trinh V.T., Schaefer S.L., Burt M., Klabunde B., Bobkova E., Kushwaha M., Foroughijabbari Y. (2023). Cell-free biosynthesis combined with deep learning accelerates de novo-development of antimicrobial peptides. Nat. Commun..

[57] Das P., Sercu T., Wadhawan K., Padhi I., Gehrmann S., Cipcigan F., Chenthamarakshan V., Strobelt H., dos Santos C., Chen P.-Y. (2021). Accelerated antimicrobial discovery via deep generative models and molecular dynamics simulations. Nat. Biomed. Eng..

[58] Lin T.-T., Yang L.-Y., Lin C.-Y., Wang C.-T., Lai C.-W., Ko C.-F., Shih Y.-H., Chen S.-H. (2023). Intelligent De Novo Design of Novel Antimicrobial Peptides against Antibiotic-Resistant Bacteria Strains. Int. J. Mol. Sci..

[59] Boone K., Wisdom C., Camarda K., Spencer P., Tamerler C. (2021). Combining genetic algorithm with machine learning strategies for designing potent antimicrobial peptides. BMC Bioinform..

[60] Wang B., Lin P., Zhong Y., Tan X., Shen Y., Huang Y., Jin K., Zhang Y., Zhan Y., Shen D. (2025). Explainable deep learning and virtual evolution identifies antimicrobial peptides with activity against multidrug-resistant human pathogens. Nat. Microbiol..

[61] Bobde S.S., Alsaab F.M., Wang G., Van Hoek M.L. (2021). Ab initio Designed Antimicrobial Peptides Against Gram-Negative Bacteria. Front. Microbiol..

[62] Zervou M.A., Doutsi E., Pantazis Y., Tsakalides P. (2024). De Novo Antimicrobial Peptide Design with Feedback Generative Adversarial Networks. Int. J. Mol. Sci..

[63] Cao J., Zhang J., Yu Q., Ji J., Li J., He S., Zhu Z. (2024). TG-CDDPM: Text-guided antimicrobial peptides generation based on conditional denoising diffusion probabilistic model. Brief. Bioinform..

[64] Vila J., Moreno-Morales J., Ballesté-Delpierre C. (2020). Current landscape in the discovery of novel antibacterial agents. Clin. Microbiol. Infect..

[65] Ageitos J.M., Sánchez-Pérez A., Calo-Mata P., Villa T.G. (2017). Antimicrobial peptides (AMPs): Ancient compounds that represent novel weapons in the fight against bacteria. Biochem. Pharmacol..

[66] Scannell J.W., Blanckley A., Boldon H., Warrington B. (2012). Diagnosing the decline in pharmaceutical R&D efficiency. Nat. Rev. Drug Discov..

[67] Schneider G., Clark D.E. (2019). Automating drug discovery. Nat. Rev. Drug Discov..

[68] Walters W.P., Murcko M.A. (2020). Assessing the impact of generative AI on medicinal chemistry. Nat. Biotechnol..

[69] Schneuing A., Harris C., Du Y., Didi K., Jamasb A., Igashov I., Du W., Gomes C., Blundell T.L., Lio P. (2024). Structure-based drug design with equivariant diffusion models. Nat. Comput. Sci..

[70] Jumper J., Evans R., Pritzel A., Green T., Figurnov M., Ronneberger O., Tunyasuvunakool K., Bates R., Žídek A., Potapenko A. (2021). Highly accurate protein structure prediction with AlphaFold. Nature.

[71] Zhao F., Qiu J., Xiang D., Jiao P., Cao Y., Xu Q., Qiao D., Xu H., Cao Y. (2024). deepAMPNet: A novel antimicrobial peptide predictor employing AlphaFold2 predicted structures and a bi-directional long short-term memory protein language model. PeerJ.

[72] Tosh C., Tec M., White J.B., Quinn J.F., Ibanez Sanchez G., Calder P., Kung A.L., Dela Cruz F.S., Tansey W. (2025). A Bayesian active learning platform for scalable combination drug screens. Nat. Commun..

[73] Proietti M., Ragno A., La Rosa B., Ragno R., Capobianco R. (2024). Explainable AI in drug discovery: Self-interpretable graph neural network for molecular property prediction using concept whitening. Mach. Learn..

